# Sedentary Behavior at Work and Cognitive Functioning: A Systematic Review

**DOI:** 10.3389/fpubh.2018.00239

**Published:** 2018-08-31

**Authors:** Valentin Magnon, Guillaume T. Vallet, Catherine Auxiette

**Affiliations:** ^1^Université Clermont Auvergne, UFR de Psychologie, Sciences Sociales, Sciences de l'Éducation, Clermont-Ferrand, France; ^2^Université Clermont Auvergne, CNRS, LaPSCo, Clermont-Ferrand, France

**Keywords:** sedentariness, sedentary behavior, cognition, work, cognitive functioning

## Abstract

**Background:** It is now well-established that sedentarity has a negative impact on the physiological functioning and health of humans, whereas very little is known about the psychological repercussions, especially in cognitive functioning. Yet, studying the cognitive effects of the sedentary lifestyle is particularly relevant in the short term for productivity and in the long term for cognitive health (accelerated aging). This systematic review therefore aims to make an inventory of the potential cognitive effects of sedentarity at the workplace.

**Methods:** Pubmed, PsycINFO, Cochrane, Web of Science, and Scopus were searched for English-language peer-reviewed articles published between January 1, 2000 and December 31, 2017 to identify studies including sedentary behavior and objective measures from cognitive domains (cognitive inhibition, cognitive flexibility, working memory, etc.). To carry out this systematic review, the 3 keywords “Sedentary” and “Cognition” and “Work” (and their derivatives) had to appear in the title or in the summary of the paper.

**Results:** Of the 13 papers that met the inclusion criteria, 9 were short-term interventions, 3 medium-term interventions, and 1 long-term intervention. Nine of them reported non-significant results. Two studies study reported deterioration in cognitive performance. Two reported an improvement in performance in cognitive tasks with one study with overweight adults and the only one study with a long-term intervention. However, these studies intend to reduce sedentary behavior, but do not allow answering the question of the potential cognitive effects of the sedentary lifestyle.

**Conclusion:** These data suggest that sedentary behavior is not associated with changes in cognitive performance in interventions that intend to reduce sedentary behavior. Then, and given the trend toward increased time in sedentary behavior, long-term prospective studies of high methodological quality are recommended to clarify the relationships between sedentary behavior and the cognitive functioning. Our systematic review identifies also the need for retrospective, longitudinal, or epidemiologic studies. It also recognizes the need to standardize methodology for collecting, defining, and reporting sedentary behavior and the need to standardize the cognitive tests used. The relationship between sedentary behavior and cognitive functioning remaining uncertain, further studies are warranted for which 8 recommendations are proposed.

## Introduction

Humans' way of life has changed dramatically over the millennia. Originally a nomadic species, then hunter-gatherers, most humans are now fixed in one place for life. This physical anchorage is also found in daily behavior. Humans have become sedentary. Among the distribution of activities in a typical day (excluding sleeping), the time spent at work is of the greatest significance. It is therefore particularly relevant to study the effects of sedentarity at work, especially for occupations that involve sitting at an office (1). Moreover, while the impact of sedentarity on health is well established (2, 3), its effects on the cognition remain poorly understood (4). The objective of this systematic review is thus to identify the effects of sedentarity at work on cognition.

A sedentary lifestyle has become the default modern lifestyle in most societies. Currently, a sedentary behavior is defined by “any waking behavior characterized by an energy expenditure ≤1.5 metabolic equivalent of task (METs), while in a sitting, reclining or lying posture” (5). Nonetheless, stationary standing, which is often associated with an energy expenditure <1.5 METs, does not produce the same effects as prolonged sitting on human physiology (Magnon et al., in revision). Indeed, sitting has negative effects on postprandial glycemic metabolism (6) resulting in a decrease in lipoprotein lipase enzyme activity (7), which causes a reduction in triglyceride hydrolysis and a decreased glucose evacuation. On the other hand, standing allows a reduction of postprandial glucose and insulin; it is therefore sufficient to get up regularly (e.g., every 20 min) or to work standing (8–11) to avoid these effects. Consequently, standing cannot be considered as a sedentary behavior even if in the past it has been categorized as such (12).

Strictly sedentary behaviors, including sitting, are recognized for their negative effects on health in the medium and long term. They increase the probability of developing type II diabetes (13), cardiovascular diseases (14), musculoskeletal disorders (MSD) (15), and even some cancers (breast, colon, colorectal, endometrial, epithelial ovarian) (2). Although some of the deleterious effects of sedentary behaviors on physical health are becoming better understood, their psychological consequences are much less so, especially on cognitive functioning. Cognition can be defined as the operations of the human mind and the mental processes that process environmental information, reasoning, thinking, problem-solving, and decision-making. Yet, some data suggest that a sedentary lifestyle may have deleterious consequences on cognition (16).

This hypothesis is supported by embodied cognition approaches that define cognitive functioning as directly grounded in the body and in the current situation (17–19). With a particular interest in sedentary issues, researchers using these approaches have shown that body posture influences the mood of individuals [sad or depressive patients tend to walk slowly and adopt a stooped posture (20)]. More importantly, the amount of energy resources available to a body of an individual changes their perception of the world (21). Tired individuals perceive a hill as being steeper than tired individuals who have just consumed a sweet drink (22). The importance of the body in cognitive functioning is also evident in studies of physical activity. Indeed, regular physical activity has a beneficial impact on cognition (23, 24), mainly on executive functioning (25–27). Executive functions refer to high-level cognitive functions and control processes that occur when the usual courses of action are no longer relevant in a given context (i.e., new, unfamiliar, dangerous, or conflicting situations), thus allowing adaptation of the individual to new situations. Beneficial effects of physical activity are also reported on working memory tasks (28) and at information-processing speed tasks (26, 29). These effects are also reported in normal aging (23, 30), which suggest that physical activity may be a protective factor against aging both in terms of physiological and cognitive functioning.

Accordingly, studies on the effects of sedentarity, outside the context of work, have shown potential negative consequences (16, 31). For instance, time spent watching television is associated with poorer episodic memory capacity (immediate and delayed recall) (4), verbal fluency (4), executive functioning (32), working memory (33), cognitive inhibition (34), and information-processing speed (34) over the long term. These results are extended to children (35) and elderly adults (36). In addition, the amount of objective sedentary behaviors (as measured by the use of accelerometers) and cognitive abilities (37) was found in a longitudinal study (over 2 years) in elderly adults (38). A large cohort study comprising adults aged 37–73 years (31) found a negative association between the amount of self-reported sedentary behaviors vs. working memory and speed of information processing. However, the potential detrimental effects of sedentarity on cognition is not always found [see meta-analysis (39)]. Moreover, it is important to take into account the type of sedentary activity, since time spent watching television and time spent reading (or listening to reading) causes different cognitive effects in young children (40). These correlational studies, outside the context of work, provide initial evidence in favor of the hypothesis that sedentarity has deleterious effect on cognition. Yet, these results are observed for long term sedentary behaviors. It is thus impossible to make a causal link between the production of sedentary behaviors and cognitive alterations since many other lifestyle habits may be involved.

There is also no evidence that sedentary behavior could impact cognition in the short-term. As the consensual definition of a sedentary lifestyle is limited at a specific moment [energy consumption ≤ 1.5 METs, (5)], it is very unlikely able to capture the potential deleterious consequences of a sedentary lifestyle on cognition. Indeed, in the field of physical activity, regular and prolonged activity is mandatory to observe beneficial effects on different cognitive domains (24, 41–46). In the same way, sedentarity may therefore have little effect on cognition at a specific moment, but only have significant consequences in the longer term. It therefore appears important to distinguish the short term and the long term when the potential cognitive effects of a sedentary lifestyle are considered. It would then be particularly relevant to consider a definition of sedentarity that is not solely “physiological” and makes possible to differentiate sedentary behavior from an individual, or from a sedentary lifestyle (36, 38).

Finally, very few studies are conducted in the context of work (47) whereas sitting for a prolonged period at work is associated with an increased risk of mortality (48). Sedentariness might thus represent a major health issue at the workplace (49), especially in the service industry where workers may remain seated 9–11 h a day (50), and may also be a barrier to efficiency and productivity at work (51, 52). Furthermore, the professional context is an environment in which it is easier to intervene to reduce sedentary behavior since a company can offer standing workstations at relatively low cost and encourage employees to get up regularly. The purpose of this systematic review is then to determine whether sedentarity could impact the cognition of an individual in the context of work.

## Methods

### Research strategies

The Preferred Reporting Items for Systematic reviews and Meta-Analyses (PRISMA) guidelines (53) were used to conduct the research and then to report the data of this systematic review. Published studies on the association between sedentarity and cognition and work were identified and cross-checked by 2 reviewers through a systematic search of the Pubmed, PsycINFO, Cochrane, Web of Science, and Scopus databases. An email alert has also been set up to warn researchers for new articles which might be published online. Articles cited in the selected articles, but not appearing in the databases searched, were also taken into account if they met the eligibility criteria. For this research, articles published between January 1, 2000 and December 31, 2017 were selected. The choice of this time window was motivated by 3 reasons: (1) The study of the sedentary lifestyle has been gaining momentum in recent years; (2) In recent studies, sedentarity is often measured objectively (use of accelerometers), whereas in older studies, it is mainly measured via the use of questionnaires (self-reported measures). In general, participants underestimate the amount of time they spend sedentary (54, 55) because they do not include all the situations they sit in (e.g., watching TV, using a computer, driving, eating); finally, (3) only recent articles distinguish between sedentarity and physical inactivity since sedentarity seems to be an independent factor of physical activity (56), as it has specific health effects (13) independent of those of physical activity (57–61).

### Study selection

To perform this systematic review, the 3 keywords “sedentary” and “cognition” and “work” (and their derivatives) were required to appear in the title or summary (see Table 1)^1^. To these 3 keywords were associated, when possible, the filters “English language,” “studies on humans,” “randomized studies,” “academic journals,” “between 2000 and 2017,” “individuals over 18 years.” The choice of the age group (over 18 years) excludes studies with children, but includes persons over 60 [the age at which an individual is considered elderly according to the WHO (63)] that are still engaged in a professional activity. The interest in studies on older people who are still likely to work rests on the hypothesis that a sedentary lifestyle could have an impact on cognitive aging.

**Table 1:** Search strategies applied in order to select studies.

**PubMed**	**PsycInfo**	**Cochrane**	**Scopus**	**Web of science**
Cognit^*^ AND Sedentary AND Work^*^	Cognition (Explode) AND Sedentary (Major Concept)	Cognit^*^ AND Sedent^*^ AND Work^*^ (Title/Abstract)	Cognit^*^ AND Sedent^*^ AND Work^*^ (Title/Abstract)	Cognit^*^ AND Sedent^*^ AND Work^*^ (Title/Abstract)
Cognit^*^AND Sedentary AND Work	Cognition (Explode) AND Sedentary AND Work	Cognition AND sedentary AND work^*^	Cognition AND sedentary AND work^*^	Cognition AND sedentary AND work^*^
Cognit^*^ AND Sedentary (Title/Abstract)	Cognit^*^ AND Sedentary AND work^*^	Cognit^*^ AND Sedentary AND Work^*^	Cognit^*^ AND Sedentary AND Work^*^	Cognit^*^ AND Sedentary AND Work^*^

### Results of the search

**Figure 1:**
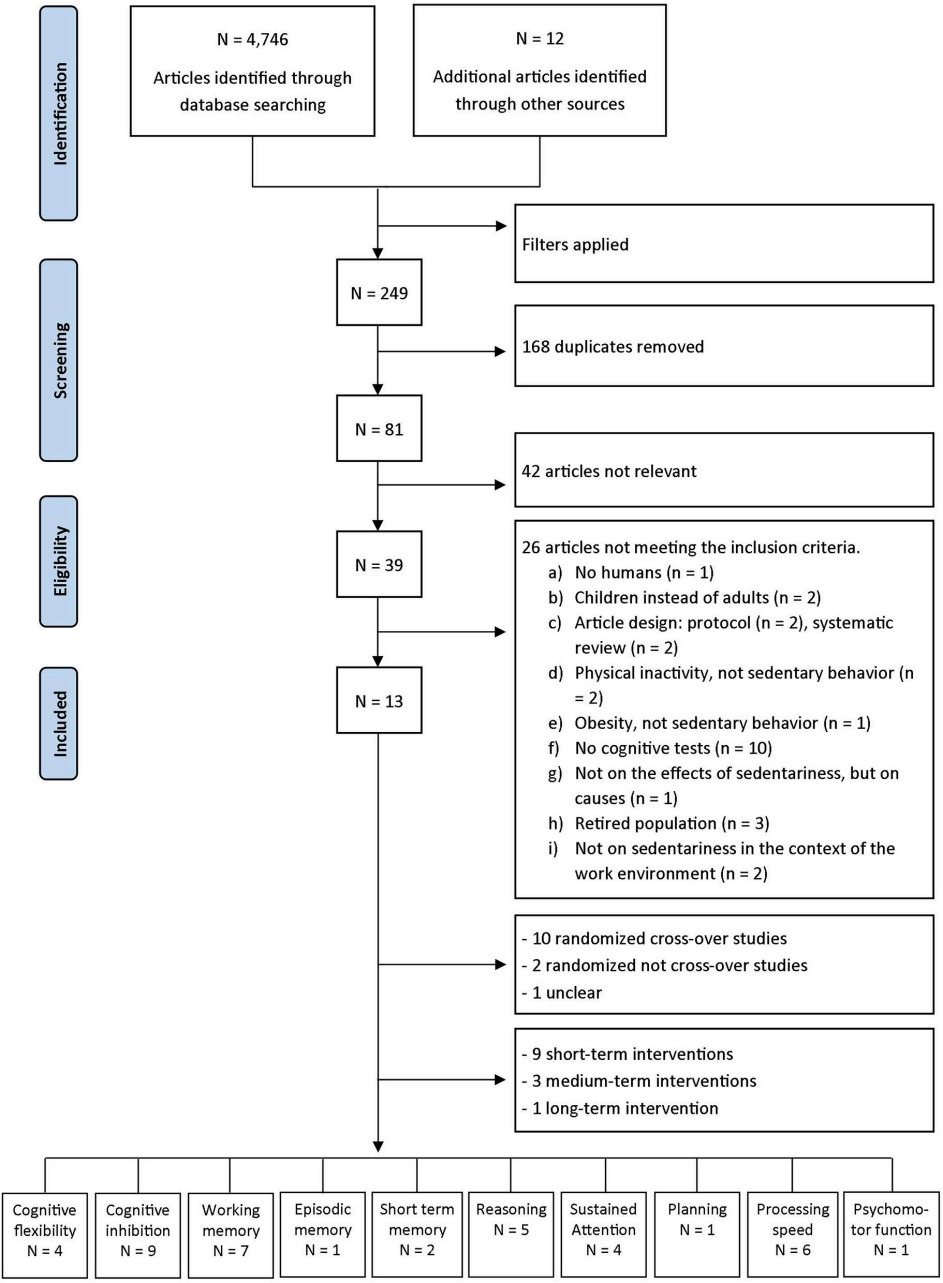
Flow chart of the steps followed in the systematic review (several cognitive tests may have been used in the same article).

From this research, 4,758 articles were obtained, including 12 from the email alert. After applying the filters, 249 articles were selected, from which 168 duplicates were removed. Of the remaining 81 articles, 42 were not related to the research problem. Of the 39 remaining articles, 26 were not retained because, despite the filters used, one article was about mice, two were about children, two were protocols, two were systematic reviews, two did not take into account the distinction between sedentarity and physical inactivity, one was more interested in the effects of obesity than those of sedentary lifestyles, 10 tested the effectiveness of interventions aimed at reducing sedentary behavior without their effects on cognition, one focused on the antecedents of sedentarity and not on its effects, three were related to retired older adults, and two did not specifically deal with work. Finally, 13 articles remained for the present review (see Figure 1).

### Data analysis

In order to determine the methodological quality and validity of the collected studies, the STROBE guidelines (Strengthening the Reporting of Observational Studies in Epidemiology) were used. In addition, the type of population (adults aged 18 and above, and seniors aged 60 and above) were identified. Finally, the experimental intervention or manipulation and the different measurements carried out were noted.

## Results

Of the 13 articles included in this review, 10 are randomized cross-over studies (64–73), 2 are randomized not cross-over studies (74, 75) and 1 unclear (76) (see Figure 1). The 13 studies selected were divided into three categories: (1) the “short-term” category includes those for which the intervention was performed at one time (64–70, 73, 74); (2) the “medium-term” category includes those for which the intervention took place over several days or weeks (71, 72, 75); and (3) the “long-term” category includes the one for which the intervention took place over several months (76). The characteristics of the selected studies are summarized in Table 2.

**Table 2:** Characteristics of selected studies.

**Articles and study design**	**Stobe score**	**Mean age (SD) [*Range*]**	**Population**	**Intervention/*Cognitive functions tested***	**Type of intervention**	**Sedentariness: type of measures/*Definition***	**Main results**
**SHORT-TERM STUDIES**							
Alderman et al. (67) Randomized cross-over	23	21.06 (1.6) (17–24)	Undergraduate students. *N* = 66	2 conditions: Treadmill-desk *vs*. Seated condition. Cognitive inhibition/Working memory	Short-term intervention: 2 conditions separated by 48 h.	NA.	No differences.
Bergouignan et al. (64) Randomized cross-over design	25	30 (5.6) (24–49)	Sedentary adults. *N* = 30	3 conditions: 6 h of uninterrupted sitting (SIT) vs. SIT plus 30 min of moderate-intensity treadmill walking vs. SIT plus six hourly 5-min microbouts of moderate-intensity treadmill walking. Cognitive flexibility/Cognitive inhibition	Short-term intervention: 1 condition per day (about 10 h).	Physical activity and sedentariness measured by a questionnaire [*International Physical Activity Questionnaire*, (77)] and by an accelerometer worn for 1 week. Sedentary if participants self-report sitting more than 9 h/day.	No differences.
Commissaris et al. (74) Randomized repeated measures design	24	29 (12) [>18]	Adults. *N* = 15	6 conditions: Treadmill desk vs. Elliptical trainer vs. Bicycle ergometer (2 conditions) vs. Standing workstation *vs*. Standard sitting position. Cognitive inhibition/Working memory/Information processing speed	Short-term intervention: 1 condition per full working day (7/8 h).	Physical activity practiced self-reported.	No differences.
Ehmann et al. (73) Randomized cross over	24	**Young adults**: 20.6 (2.0) (17–27) **Middle-aged adults**: 45.6 (11.8) (30–64)	Young adults: *N* = 32 Middle-aged adults: N = 26	2 conditions: Treadmill walking (low intensity) vs. Seated control condition. Cognitive flexibility/Cognitive inhibition/Working memory/Reasoning	Short term intervention: 2 experimental conditions, each separated by at least 48 h.	Physical activity self-reported [*physical activity readiness questionnaire*, (78)] and measured by an accelerometer wore during test sessions.	No differences.
John et al. (68) Randomized cross-over	22	26.4 (4.04) [NC]	Graduate students. *N* = 20	2 conditions: Treadmill desk vs. Sitting. Cognitive inhibition/Reasoning/Information processing speed	Short-term intervention: 2 visits of 60 minutes separated by 2 days; 1 condition/day.	NA.	Poorer performances on reasoning and information processing speed in the treadmill desk condition.
Ohlinger et al. (65) Randomized cross-over	20	43.2 (9.3) (22–59)	Employees of Miami University. *N* = 50	3 conditions: Sitting vs. Standing vs. Walking. Cognitive inhibition/Short term memory/Information processing speed	Short-term intervention: A single 75-min visit.	Physical activity and sedentariness measured by questionnaire (hours spent sitting at work each day, and number of days they exercise each week).	No differences other than a decrement on the processing speed task during walking compared to sit and stand.
Pilcher and Baker (69) Randomized cross-over	21	19.64 (1.05) [NC]	Undergraduate students. *N* = 38	2 conditions: Cycling vs. Sitting at a traditional desk. Reasoning	Short-term intervention: Two 45-min sessions separated by 24 h at least.	NA.	No differences.
Schwartz et al. (66) Randomized cross-over	25	25.4 (3.3) (19–31)	Students. Control group (*n* = 15) Experimental Group (*n* = 30) *N* = 45	Control group: Sitting for 5 consecutive 30-min trials each. Experimental group, 2 conditions: Alternate sitting and standing postures every 30 min. 5 times *vs*. sit for 5 trials. Cognitive inhibition/Sustained attention/Information processing speed	Short-term intervention: 2 whole days separated by 7 days; 1 condition per day.	Physical activity and sedentariness measured by a questionnaire [*International Physical Activity Questionnaire*, (77)].	No differences.
Torbeyns et al. (70) Randomized cross-over	23	35.7 (10.3) [NC]	Adults with a sedentary occupation. *N* = 23	2 conditions: Cycling desk vs. Sitting on a conventional chair. Cognitive inhibition/Episodic memory/Sustained attention/Information processing speed	Short-term intervention: 2 visits separated by 1 week; 1 condition per visit.	Physical activity practiced self-reported [*International Physical Activity Questionnaire*, (77)]. Sedentariness if the individual is seated for at least 70% of the workday.	No differences.
**MEDIUM-TERM STUDIES**							
Edwards and Loprinzi (75) Randomized controlled, parallel group intervention	27	21.74 (2.82) (17–34)	Adults. Control group (*n* = 10). Experimental group (*n* = 23). *N* = 33	Control group: Normal practice of physical activity. Experimental group: Reduce physical activity as much as possible for a week. Working memory/Reasoning/Sustained attention/Planning	Medium-term intervention: reduction of physical activity during 1 week.	Physical activity practiced and sedentariness self-reported by a questionnaire [*International Physical Activity Questionnaire*, short form, (77)] and wearing an accelerometer for 1 week prior to the intervention and wearing a pedometer during 7 days. Sedentariness if no practice of structured physical activity and less than 5,000 steps a day.	No differences.
Mullane et al. (71) Randomized cross-over full-factorial design	24	30 (15) (17–57)	Overweight adults with a sedentary office-based occupation. N = 9	4 conditions: Sit vs. Sit-Stand *vs*. Sit-Walk *vs*. Sit-Cycle. Working memory/Reasoning/Psychomotor	Medium-term intervention: Each condition performed across 4 consecutive weeks, 7 days apart.	Physical activity and sedentariness measured by an accelerometer.	Improved working memory, reasoning and psychomotor if short moments of light physical activity (standing, walking, cycling).
Russell et al. (72) Randomized cross-over	24	40.08 (11.93) (21–61)	Employees of the university of Tasmania. *N* = 36	2 conditions: Sitting vs. Standing (or the reverse) for 1 h per day for 5 consecutive days. Working memory/Sustained attention/Information processing speed/Cognitive flexibility/Cognitive inhibition/Short-term memory	Medium-term intervention: 1 h/day for 5 consecutive days.	Physical activity and sedentariness measured by a questionnaire [Occupational Sitting and Physical Activity Questionnaire, (79)] and by an accelerometer.	No differences.
**LONG-TERM STUDY**							
Fanning et al. (76) Unclear	25	65.4 (4.6) (59–78)	Older people among whom 120 still have a professional activity. *N* = 247	3 conditions: Substituting 30 min of sedentary behavior with 30 min of (a) light activity, (b) moderate-to-vigorous physical activity, or (c) sleep. Cognitive flexibility/Working memory	Long-term intervention: during 6 months.	Sedentariness measured by accelerometer during 7 consecutive days. Sedentariness if number of counts per minute at the accelerometer is < 50.	Better performances.

*NA, Not Available; sedentarity was not defined, and not assessed or reported*.

### Measurement of sedentarity

The presence of a clear and accepted definition of a sedentary lifestyle was sought in the articles to verify the absence of confusion between sedentarity and physical inactivity. However, only 3 articles define sedentarity (71, 74, 76) [all behaviors resulting in energy expenditure ≤ 1.5 METs (5)]. For 2 articles (64, 70), the standard of sedentarity was to remain seated for a long time during the day. In 2 others articles they used a definition based on the accelerometers (75, 76). Finally, for 8 articles (65–69, 71–74), the criteria for sedentary were not indicated (see Table 2).

Sedentarity was assessed by accelerometers and self-reported measures in four studies (64, 72, 73, 75), only by self-reported measures in 4 studies (65, 66, 70, 74) and only by accelerometers in 2 studies (71, 76). For the last 3 studies (67–69), sedentarity was neither assessed nor reported. Solely accelerometers allow an objective and sensitive measurement of the amount of sedentary behavior.

### Cognitive functions tested

The cognitive functions tested were cognitive flexibility (64, 72, 73, 76), cognitive inhibition (64–68, 70, 72–74), working memory (67, 71–76), episodic memory (70), memory short-term (65, 72), reasoning (68, 69, 71, 73, 75), sustained attention (66, 70, 72, 75), planning (75), information processing speed (65, 66, 68, 70, 72, 74), and psychomotor function (71) (see Table 3). Most of the functions tested involve attentional processing and/or executive control. This choice is probably justified by the fact that physical activity preferentially improves these functions (23, 41, 111, 112).

**Table 3:** Cognitive functions measured, and tests used in the selected articles.

**Cognitive functions**	**Tests**	**Authors**
Cognitive flexibility	Trail Making Test (TMT) (80)	(64, 72)
	Wisconsin card sorting test (81)	(73)
	Task switching paradigm (82)	(76)
Cognitive inhibition	Stroop and their derivatives (83)	(65–68, 70, 72, 73)
	Go-No-Go (84)	(74)
	Flanker task (85)	(64, 67, 74)
Working memory	N-back (86)	(71, 74)
	Spatial span (87)	(75)
	Digit span subtest	(72)
	Sternberg working memory task (88)	(73)
	Scholastic assessment test (SAT) (89)	(67)
	Paired associates (90)	(75)
	Spatial working memory task (91)	(76)
	Letter Number Sequencing subtest (LNS) (92)	(72)
Episodic memory	Rey auditory verbal learning test (93)	(70)
Short term memory	The auditory consonant trigram test (94)	(65)
	Digit span subtest (92)	(72)
Reasoning	Grammatical reasoning (95)	(75)
	Graduate record examination (96)	(68)
	Tower of London (97)	(73)
	Set-shifting test (98)	(71)
	Odd one out (95)	(75)
	Law School Administration Test (LSAT) (99)	(69)
	Raven's standard progressive matrices (100)	(69)
Sustained attention (concentration)	Feature match (101)	(75)
	Polygon (90)	(75)
	Four-choice visual reaction time test (CRT) (102)	(72)
	d2R (103)	(66)
	Rosvold Continuous Performance Test (RCPT) (104)	(70)
Planning	Spatial search (105)	(75)
	Spatial slider (106)	(75)
Information processing speed	Typing task (107)	(68, 70, 74)
	Fast counting task (108)	(74)
	The Digital Finger Tapping test (DFTT) (109)	(65)
	Digit Symbol Coding subtest (DSC) (92)	(72)
	Trail Making Test (TMT) (reaction time) (110)	(72)
	Stroop (reaction time) (83)	(70)
	Rosvold Continuous Performance Test (reaction time) (104)	(70)
	Transcription test	(70)
	Text editing task	(66)
Psychomotor function	The detection test (98)	(71)

### Age of participants

On the 13 articles selected, 9 studies were conducted among adults aged 18–50 (64, 66–70, 73–75), 1 with adults aged 18–58 (71), 1 with adults aged 23–60 (65) or between the ages of 22 and 62 (72). Finally, 1 study was conducted among people aged between 60 and 79 years (76). In 2 studies, the range of the age's participants was homogeneous (66, 75) and in 5 other studies age was controlled as a co-variable (65, 67, 71, 73, 76).

### Main results

In the selected studies, two types of paradigms were used: (1) one was to compare the achievement of a cognitive task either sedentarily (sitting in a traditional office) or while performing or just after completing light or moderate physical activity (through the use of dynamic workstations or while working standing) (64–74); (2) the other compared cognitive performance with different tests of physically active individuals to that of sedentary individuals and without using a dynamic workstation during testing (75, 76).

#### Studies involving the use of dynamic workstations

Adults working on a treadmill desk (64, 65, 67, 68, 70, 73, 74), or on an elliptical trainer (74), or on a cycling desk (69, 70, 74) do not perform better than those working at a traditional desk, whether for tasks of cognitive inhibition (64, 65, 67, 68, 70, 73, 74), speed of information processing (70, 74), working memory (67, 73, 74), episodic memory (70), short-term memory (65), sustained attention (70), cognitive flexibility (64, 73), or reasoning (69, 73). Similarly, when the participants have to alternate between sitting and standing (66, 72), no difference is observed with tasks of cognitive flexibility, cognitive inhibition, working memory, short memory, sustained attention, and speed of information processing. Reversely, adults working at a treadmill desk perform worse on reasoning (68) and processing speed (65, 68) tasks than adults working at a conventional desk, but after short periods of physical activity (e.g., walking, standing, pedaling), overweight adults perform better on a working memory, psychomotor and reasoning tasks than when they sit without physical activity (71).

#### Studies that do not involve the use of dynamic workstations

Replacing sedentary behaviors for 6 months through moderate physical activity in older people improve their performance at working memory and cognitive flexibility tasks (76). Using a reverse principle, adults forced to remain inactive for a week do not show modified performance on working memory, reasoning skills, planning skills, or concentration (75).

## Discussion

The purpose of this systematic review was to identify the potential effects of work-related sedentariness on cognitive functioning. While the effects of sedentarity on physical health are now established (2, 3, 113), the impact on psychological health and cognitive abilities remains uncertain (16). Increasing sedentary behavior at the workplace is a major public health issue and a particularly relevant choice because of (1) the importance of time spent working per day; (2) the possibility of controlling this environment (and therefore intervening for workers); and (3) the economic and health implications of possible cognitive changes due to sedentary productivity (in the short term) and the risk of cognitive decline (in the long term) of the workers concerned. This distinction is equivalent to seeking the effects of sedentary behavior regardless of an individual's lifestyle (≤1.5 METs at a given time), a predominantly sedentary lifestyle (36, 38). Thus, as highlighted in the introduction, we should distinguish on the one hand longitudinal or correlational studies that are intended to determine the existence and factors of the possible repercussions of sedentarity on cognitive health, and on the other hand, studies and interventions whose objective is the reduction of sedentary behaviors. The filters applied for this systematic review resulted in only interventional studies. Among them, it seems still relevant to distinguish the studies according to the duration of the intervention: in the short term, medium term, and long term.

### Summary of the main results

The results, taken as a whole, appear contradictory. Four studies (65, 68, 71, 76) among the 13 identified highlight a significant change in cognition related to sedentary behavior. Of these 4 studies, 2 (71, 76) show an improvement in cognitive performance when sedentary behavior is decreased, but two shows deterioration (65, 68).

These contradictions do not seem to be explained by the type of intervention employed. Of the 11 studies involving the use of a workstation (64–74), 8 (64, 66, 67, 69, 70, 72–74) found no alteration of cognitive functions, one (71) reports an improvement in performance to a task of working memory, psychomotor functions and reasoning, while the last 2 (65, 68) report a drop in performance at a task of reasoning (68) and speed of information processing (65, 68) when using a treadmill desk (but not at a task of cognitive inhibition). The cognitive function considered also does not seem to be able to explain these contradictions, since, out of all the studies included, 2 report beneficial effects on working memory (71, 76) while others report no effect on the same function (67, 72, 74, 75).

The distinction between short-term, medium-term and long-term intervention, on the other hand, offers a different interpretation. No short-term and medium-term interventions report a significant improvement in cognitive functioning when measures are taken to decrease sedentary behavior, with the exception of one study that targets overweight individuals, thus limiting possible generalization of this result (71). On the contrary, two of the short-term interventions show a decrease in performance (65, 68); these studies were the only two to not offer a familiarization session that allowed participants to adapt to the use of the dynamic workstation. This result could then be explained by a dual-task situation (114). The results reported by these two studies are a decrease in performance at reasoning tasks (68) and speed of information processing (65, 68) but not for tasks of cognitive inhibition (65, 68) and memory in the short term (65) when using a treadmill at the same speed (1.6 km/h). It is therefore possible that this dual-task situation affects information processing and reasoning tasks because the former could be more costly with regard to motor skills and the second cognitively more costly than the other tasks. The only long-term study identified in this systematic review (76), however, suggests a beneficial effect on the cognitive functioning of people with a less sedentary lifestyle and work.

Among the few cognitive functions tested, significant results are observed for working memory (71, 76), reasoning (71), psychomotor function (71), and mental flexibility (76). These observations are consistent with the effects of physical activity on cognition (23, 24, 41, 82).

### Explanatory hypotheses of divergent results

These seemingly contradictory results lead us to consider 5 factors to be taken into account in the study of the possible effects of sedentarity on cognition.

1. **Duration of the intervention or duration of the sedentarity**. These durations should be controlled as the effects of sedentarity on cognition may stem from the chronic processes observable only over the long term.
2. **Daily physical activity**. Regular physical activity may be sufficient to have a protective effect on cognitive functioning, making the effects of sedentary lifestyles invisible (75).
3. **Testing time**. The timing of the testing, i.e., during physical activity, immediately after or in the longer term appears to impact differently the results. Testing cognitive functions during physical activity may test more divide attentional abilities than not being sedentary (114) especially when no familiarization session is provided [see (65, 68)].
4. **Age of the participants**. Chronic sedentary effects are more likely to be apparent in older individuals than in younger individuals. In addition, as advancing age is associated with cognitive decline (115), the effects potentially observed in older sedentary individuals must be age-controlled (matched control group) as it is the case for only 7 of the 13 studies included here (65–67, 71, 73, 75, 76).
5. **Measure of sedentarity**. How sedentarity is measure may impact the results because subjective measures (questionnaires) [see (83, 86, 111, 112)] may underestimate the amount of time spent sedentarily (54, 55).

### Recommendations

This systematic review of the literature has highlighted the lack of studies on the consequences of sedentariness on cognitive functioning at work. The data mainly not showed any significant results. Nevertheless, such a link is predicted by embodied cognition approaches (18, 19, 21) and is supported by studies of the effects of physical activity on cognition (23, 24). It would seem, then, that the chronicity of the behaviors is the determining factor. To answer these problems, it appears essential to follow various recommendations. A first action would consist in determining if sedentary behaviors can have an impact on cognitive functioning. To do this, retrospective, longitudinal, or epidemiological studies should be conducted. These studies should propose: (1) questionnaires or objective measures assessing the importance, frequency and duration of sedentary behavior, making sure to distinguish whether these behaviors occur at work or not; (2) objective questions or measurements of physical activities performed; and (3) a cognitive assessment, if possible exhaustive, or at least targeting working memory, executive functions, and the speed of information processing. It would then be possible to determine to what extent sedentarity at work, in relation to sedentary life outside of work and physical activity, makes it possible to explain the cognitive functioning of an individual by controlling for age, sex, level of education, and other protective or risk factors of cognition (sleep apnea, cognitive reserve, etc.).

If the results prove significant, then it will become relevant to set up interventional studies. Beyond the fact that these studies should favor a randomized plan with a random distribution of participants in each experimental condition, they could also follow the 8 recommendations bellow organized in order of importance.

1. **Duration of the intervention**. Short interventions are ineffective in showing a positive effect on cognitive functioning, at least in the general population [but see (71) for overweight people], but they are in the long term [see (88)]. This last study is the only one to propose an intervention over 6 months. However, work on physical activity suggests that effects can be seen as early as 4 weeks of intervention (116).
2. **Baseline**. Since measuring the effectiveness of an intervention requires comparing performance before and after the intervention, it is necessary to choose tests that are not very sensitive to the test-retest effect, or to include a control group that does not benefit from any intervention.
3. **Tests**. The cognitive tests used must be valid, reliable, and sensitive. The use of standardized tests commonly recognized among researchers in cognitive psychology or neuropsychology is recommended. Moreover, and ideally, a cognitive function should be evaluated by two different measures (117). In the case of an intervention involving the use of dynamic workstations, it is important to consider when the participants are cognitively assessed (before, during or after physical activity). If the cognitive test is administered while performing physical activity, it is important to consider the degree of habituation of participants to work while doing physical activity. Familiarization sessions are therefore recommended (70).
4. **Measurement of current sedentarity**. Objective measures of sedentary behavior should be favored with, for example, the use of accelerometers (16, 38).
5. **Physical activity and previous sedentary lifestyle**. It also seems essential to assess the level of physical activity of the participants (current and previous) and the previous level of sedentarity of the participants, since they could have long-term effects on the cognitive health of individuals.
6. **Homogeneity of population**. The target population should be as homogeneous as possible to control for the possible influence of variables such as socio-economic level or age group of participants. Moreover, since the professional activity that requires a regular elaborated cognitive engagement seems to have a protective effect on cognitive decline (118), it is also recommended to take into account the activities carried out in the context of work.
7. **Specificity of effects of the intervention**. The specifics of the type of intervention should ideally be controlled to determine whether it is the intervention itself that produces an effect and not other factors combined (such as simply participating in a study or changing the season). This specificity could be tested by including a group benefiting from an intervention with no expected effect on the sedentary lifestyle (e.g., speech group).
8. **Maintaining effects over time**. Finally, it is particularly interesting to know the maintenance of the effects after the intervention, which can be done by including measures several months after the end of the intervention relating not only to the cognitive functions, but also to the maintenance of the practices used to reduce the sedentary lifestyle.

### Limitations

Several limitations must be considered when interpreting these findings. There may be a publication bias which limits generalizability of our findings; however, this limitation is inherent in all systematic reviews. Indeed, the review was limited to peer-reviewed published work and to the search terms and databases contained in our Methods section. Studies that have not been abstracted with these key words are inevitably missing from the review, but we also searched the cited works in each selected article. Our search strategy was also limited to English-only studies, which may have resulted in a language and cultural bias. In addition, the heterogeneity in methods among the studies—such as the use of different cognitive tests as well as the small sample sizes—, and the small number of papers that fulfilled the inclusion criteria, have to be accepted as necessary due to the infancy of this field of research on sedentary behavior and cognition at the workplace.

## Conclusion

Effects of work-related sedentarity on cognition appear mixed. Most of the studies do not report significant results on cognition, but other psychological consequences such as a decrease in the feeling of tiredness (64), an increase in motivation (69), and a more positive mood (64) [see also (119)] are nevertheless observed. The psychological repercussion of sedentariness may be better explored by considering sedentarity no longer through the physiological definition [≤1.5 METs, (5)], but through a psychological definition referring to the prospective cognitive consequences of this way of life (120–123). It is also important to manipulate the production of sedentary behaviors instead of the practice of activities, as it was the case in most of the included studies.

Although chronic sedentary lifestyles and physical inactivity share many similarities, the distinction between these two concepts is fundamental. Thus, intervention for physical inactivity focuses on the establishment of sports activities that is usually done during leisure time. On the other hand, interventions to combat a sedentary lifestyle do not require a sporting activity, since simply standing can be enough to counteract the physiological effects of a sedentary lifestyle (8–10). This particularity makes it possible to intervene not only on the leisure time of an individual, but also on his or her time and place of work. It seems much simpler to suggest a person to get up regularly or to work while standing than to go for a 15-min run during a break. Health preventive programs may then propose work adaptation such as broadcast a signal to encourage the workers to get up every 20 min or suggest the use of standing desks or active workstations whenever possible. Finally, more information about the consequences of sedentarity on both physical and psychological health should be available to the workers and to the structures.

## Author contributions

VM reviewed the literature, revised CA's article outline, created and maintained a Zotero database, wrote the initial abstract, manuscript, and table and figure drafts, and revised the second drafts following feedback from CA. CA reviewed the literature, and proposed an article outline. Both CA and GV contributed sections of the initial drafts, and made editorial suggestions for the second drafts. VM, CA, and GV discussed the conceptual issues and themes for the review article. All authors contributed to manuscript revision, read and approved the submitted version.

### Conflict of interest statement

The authors declare that the research was conducted in the absence of any commercial or financial relationships that could be construed as a potential conflict of interest.

## Footnotes

^1^There are articles that can be similar to an intervention against sedentarity in at the workplace, but do not include these keywords. For the sake of rigor, they were not included in this systematic review because assessments of sedentary lifestyles, cognition, and intervention were not necessarily well controlled or reported. It is noteworthy, however, that the results of these studies are in line with those reported in this systematic review [see (62) for a review of active workstations].
